# Perception and preventive actions against COVID-19 in domestic and international students

**DOI:** 10.1192/j.eurpsy.2021.850

**Published:** 2021-08-13

**Authors:** E. Nikolaev, A. Aleksandrov, I. Poverinov, L. Niyazov

**Affiliations:** 1 Social And Clinical Psychology, Ulianov Chuvash State University, Chebokasry, Russian Federation; 2 Public Law Department, Ulianov Chuvash State University, Chebokasry, Russian Federation; 3 Philosophy, Sociology And Pedagogy Department, Ulianov Chuvash State University, Chebokasry, Russian Federation; 4 Medical Chemistry Department, Bukhara State Medical Institute, Bukhara, Uzbekistan

**Keywords:** perception, prevention, COVID-19, students

## Abstract

**Introduction:**

Young people, regarded as less susceptible to the virus, may differently perceive the situation associated with the COVID-19 outbreak.

**Objectives:**

To determine the differences in perception of COVID-19 and preventive actions taken against it by domestic and international university students under the spreading threat of COVID-19 illness.

**Methods:**

During the outbreak of COVID-19, 224 domestic and 312 international students of Russian universities responded via on-line to the self-constructed Attitude towards COVID-19 Questionnaire.

**Results:**

The showings of the international students were surely higher than those of domestic students in seriousness of their evaluation of the COVID-19-related situation in the world (p=.0006), in the country (p=.0096), and in the region (p=.0390); in the evaluation of the virus-related risks for aged and chronic patients (p=.0075), in adequacy of measures taken by the government against COVID-19 (p=.0114), in degree of disturbing their customary way of life (p=.0363), and ruining their plans for the future (p=.0161). The international students, who live mostly not at their homes and have a higher stress level (p=.0227), showed higher interest to COVID-19-related news (p=.0001), they were stricter in taking preventive measures: in wearing a mask, washing hands, keeping the distance in order to reduce the risk of the virus infection (p=.0009).

**Conclusions:**

During the COVID-19 outbreak, both the international and domestic students are calm in perceiving the threat to their health and life. At the same time, with a higher stress level, the international students are more watchful concerning the situation of COVID-19 spread, and they more strictly obey the restrictive measures.

